# Anti-proliferative effects of T cells expressing a ligand-based chimeric antigen receptor against CD116 on CD34^+^ cells of juvenile myelomonocytic leukemia

**DOI:** 10.1186/s13045-016-0256-3

**Published:** 2016-03-16

**Authors:** Yozo Nakazawa, Kazuyuki Matsuda, Takashi Kurata, Akane Sueki, Miyuki Tanaka, Kazuo Sakashita, Chihaya Imai, Matthew H. Wilson, Kenichi Koike

**Affiliations:** Department of Pediatrics, Shinshu University School of Medicine, 3-1-1, Asahi, Matsumoto, 390-8621 Japan; Department of Laboratory Medicine, Shinshu University Hospital, Matsumoto, Japan; Division of Hematology/Oncology, Nagano Children’s Hospital, Azumino, Japan; Department of Pediatrics, Niigata University School of Medicine, Niigata, Japan; Department of Medicine, Division of Nephrology and Hypertension, Vanderbilt University School of Medicine, Nashville, TN USA

**Keywords:** Juvenile myelomonocytic leukemia, Chimeric antigen receptor, GM-CSF receptor, Leukemic stem cells, *piggyBac* transposon

## Abstract

**Background:**

Juvenile myelomonocytic leukemia (JMML) is a fatal, myelodysplastic/myeloproliferative neoplasm of early childhood. Patients with JMML have mutually exclusive genetic abnormalities in granulocyte-macrophage colony-stimulating factor (GM-CSF) receptor (GMR, CD116) signaling pathway. Allogeneic hematopoietic stem cell transplantation is currently the only curative treatment option for JMML; however, disease recurrence is a major cause of treatment failure. We investigated adoptive immunotherapy using GMR-targeted chimeric antigen receptor (CAR) for JMML.

**Methods:**

We constructed a novel CAR capable of binding to GMR via its ligand, GM-CSF, and generated *piggyBac* transposon-based GMR CAR-modified T cells from three healthy donors and two patients with JMML. We further evaluated the anti-proliferative potential of GMR CAR T cells on leukemic CD34^+^ cells from six patients with JMML (two *NRAS* mutations, three *PTPN11* mutations, and one monosomy 7), and normal CD34^+^ cells.

**Results:**

GMR CAR T cells from healthy donors suppressed the cytokine-dependent growth of MO7e cells, but not the growth of K562 and Daudi cells. Co-culture of healthy GMR CAR T cells with CD34^+^ cells of five patients with JMML at effector to target ratios of 1:1 and 1:4 for 2 days significantly decreased total colony growth, regardless of genetic abnormality. Furthermore, GMR CAR T cells from a non-transplanted patient and a transplanted patient inhibited the proliferation of respective JMML CD34^+^ cells at onset to a degree comparable to healthy GMR CAR T cells. Seven-day co-culture of GMR CAR T cells resulted in a marked suppression of JMML CD34^+^ cell proliferation, particularly CD34^+^CD38^−^ cell proliferation stimulated with stem cell factor and thrombopoietin on AGM-S3 cells. Meanwhile, GMR CAR T cells exerted no effects on normal CD34^+^ cell colony growth.

**Conclusions:**

Ligand-based GMR CAR T cells may have anti-proliferative effects on stem and progenitor cells in JMML.

**Electronic supplementary material:**

The online version of this article (doi:10.1186/s13045-016-0256-3) contains supplementary material, which is available to authorized users.

## Background

Juvenile myelomonocytic leukemia (JMML) is a fatal, mixed myeloproliferative, and myelodysplastic disorder that occurs in infancy and early childhood. Patients with JMML have genetic abnormalities in granulocyte-macrophage colony-stimulating factor (GM-CSF) signaling pathways, such as inactivation of *NF1* or mutations in *PTPN11*, *NRAS*, *KRAS*, and *CBL* [1, 2]. According to whole-exome sequencing, Sakaguchi et al. [3] demonstrated that *SETBP1* and *JAK3* mutations are common recurrent secondary events associated with poor clinical outcomes. In our genetic analyses of individual granulocyte-macrophage colonies, these non-RAS pathway gene mutations may represent the second genetic aberration in a proportion of JMML children with *PTPN 11* mutations [4]. Stieglitz et al. [5], using droplet digital polymerase chain reaction, detected *SETBP1* mutations more frequently in patients with JMML, indicating the possibility that subclonal mutations at diagnosis confer a dismal prognosis in JMML. More recently, Caye et al. [6] reported multiple concomitant genetic hits targeting the RAS pathway and new pathway activation involving phosphoinositide 3-kinase and the mTORC2 complex through RAC2 mutation. In addition, their study defined PRC2 loss that switches the methylation/acetylation status of histone H3 lysine 27.

Allogeneic hematopoietic stem cell transplantation is currently the only curative treatment option for JMML; however, disease recurrence is a major cause of treatment failure [7]. There have been several reports of patients being successfully treated by donor lymphocyte infusions for post-transplant relapse [8, 9], suggesting that immune-based therapies, such as T cell-mediated immunotherapy, may represent feasible treatment approaches in JMML. Nabarro et al. [10] demonstrated the generation of immunostimulatory dendritic cells from malignant JMML clones. Allogenic T cells stimulated by leukemic dendritic cells were able to lyse leukemic JMML cells; however, this anti-leukemic effect may depend on alloimmune mechanisms and fail to direct activated T cells toward leukemia-associated antigens. Thus, this treatment approach may be limited to cases of post-transplant relapse in a similar manner to donor lymphocyte infusions. In addition, infused T cells may induce severe graft-versus-host disease. Hirano et al. [11] demonstrated that γ-globin-specific cytotoxic T cells from healthy donors were capable of lysing primary JMML cells in an HLA-A2-restricted manner. Nevertheless, cytotoxic T cells were found to have no effect on cells derived from a patient with JMML who had an HbF level of 1 %. In contrast, γ-globin-specific T cells may disrupt post-transplant erythropoiesis as HbF level markedly increases following cord blood transplantation. Additionally, the critically important issue of whether JMML stem cells express γ-globin remains unclear.

Adoptive immunotherapy using chimeric antigen receptors (CAR) targeting tumor-associated antigens represents a novel approach for the treatment of hematological malignancies [12]. In particular, CD19-targeted CAR T cell therapy has achieved dramatic clinical success in pediatric patients with refractory/relapsed acute lymphoblastic leukemia [13, 14]. More recently, we developed CD19 CAR T cells using a *piggyBac* transposon system and found superior transgenic T cell-mediated lysis of Philadelphia chromosome-positive acute lymphoblastic leukemia cells regardless of the presence or absence of a *BCR-ABL* T315I mutation resistant to tyrosine kinase inhibitors [15].

In the present study, we developed a novel CAR capable of binding to the GM-CSF receptor (GMR, CD116) using ligand-receptor interactions. T cells were then modified to express the developed GMR CAR through the use of a *piggyBac* transposon system. We then examined the anti-proliferative activity of ligand-based GMR CAR T cells on JMML CD34^+^ cells.

## Results

### Generation of T cells modified to express GMR CAR with *piggyBac* transposons

**Fig. 1:**
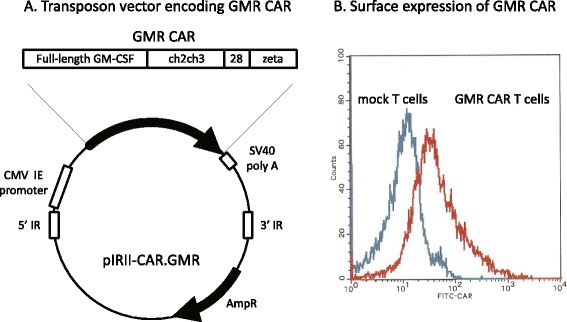
Generation of T cells modified to express GMR CAR with *piggyBac* transposons. **a** A CAR targeting GM-CSF receptor (*GMR CAR*) was constructed by replacing the anti-CD19 scFv portion of a *piggyBac* transposon plasmid encoding CD19 CAR (*pIRII- CAR.CD19*) with full-length GM-CSF. **b** Surface expression of GMR CAR in transgenic T cells expanded by stimulation with anti-CD3/CD28 mAbs

A CAR targeting CD116 (GMR CAR) was constructed by replacing the anti-CD19 single-chain variable fragment (scFv) portion with full-length GM-CSF in a *piggyBac* transposon plasmid encoding CD19 CAR (pIRII-CAR.CD19) reported previously [15, 16], as shown in Fig. 1a. A total of 1.0 × 10^7^ peripheral blood mononuclear cells (PBMCs) from approximately 10 ml of blood from healthy donors was electroporated with a GMR CAR-containing *piggyBac* transposon and the *piggyBac* transposase plasmids. Transgenic cells were then stimulated with anti-CD3/CD28 monoclonal antibodies (mAbs) in a serum-free culture system. After immunomagnetic isolation and enrichment of GMR CAR-positive cells, the cells were then re-stimulated with anti-CD3/CD28 mAbs. After 21 days of culture, a total of 5.2 ± 2.8 × 10^8^ cells were obtained from three healthy donors. Immunophenotyping of isolated cells revealed that 99.2 ± 0.6 % were positive for CD3, 18.1 ± 12.1 % were positive for CD4, and 78.3 ± 9.7 % were positive for CD8. The surface expression of GMR CAR was displayed by 40.9 ± 7.8 % of CD3^+^ cells (Fig. 1b).

**Fig. 2:**
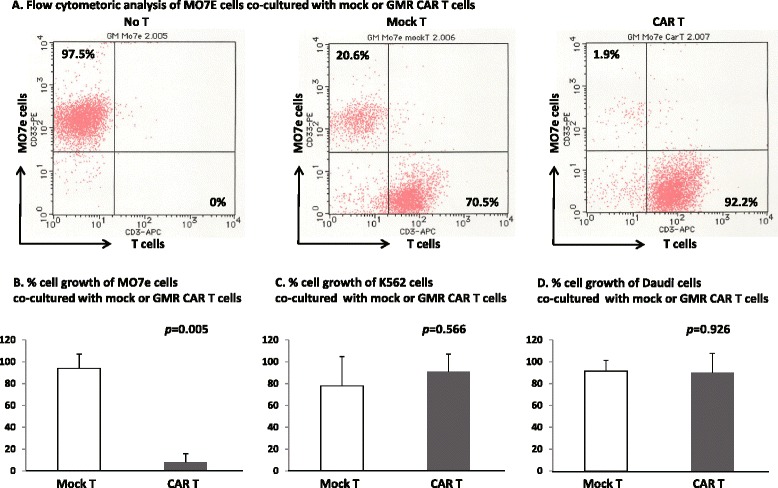
Anti-proliferative effects of GMR CAR T cells on MO7e, K562, and Daudi cells. **a** Flow cytometric analysis of MO7e cells co-cultured with mock T cells or GMR CAR T cells at an E:T ratio of 2:1. After 4 days, cultured cells were stained with either PE-conjugated anti-CD33 or PE-conjugated anti-CD19 and APC-conjugated CD3 mAbs. **b** % growth (proportion of initial cell numbers) of MO7e cells co-cultured with mock T cells or GMR CAR T cells from three healthy donors. The culture of 1 × 10^5^ MO7e cells alone generated 3.6 × 10^5^ cells in the presence of 10 ng/ml GM-CSF after 4 days. **c** % growth (proportion of initial cell numbers) of K562 cells co-cultured with mock T cells or GMR CAR T cells from three healthy donors. The culture of 1 × 10^5^ K562 cells alone generated 8.0 × 10^5^ cells after 4 days. **d** % growth (proportion of initial cell numbers) of Daudi cells co-cultured with mock T cells or GMR CAR T cells from three healthy donors. The culture of 1 × 10^5^ Daudi cells alone generated 9.4 × 10^5^ cells after 4 days

As shown in Fig. 2a, b, the addition of GMR CAR T cells from three donors at an effector to target (E:T) ratio of 2:1 diminished the numbers of MO7e cells stimulated with GM-CSF for 4 days, by 8.0 ± 7.8 % of the number of cells observed in control cultures (0.3 ± 0.3 × 10^5^ cells in the presence of GMR CAR T cells and 3.8 ± 0.4 × 10^5^ cells in the absence of GMR CAR T cells). The number of MO7e cells observed following co-culture with mock T cells at an identical ratio was 94.0 ± 13.0 % of the number of cells observed in control cultures (3.6 ± 0.2 × 10^5^ cells in the presence of mock T cells). No significant difference in the anti-proliferative effect of GMR CAR T cells on MO7e cells was observed between stimulation with GM-CSF and stimulation with stem cell factor (SCF), thrombopoietin (TPO), and interleukin (IL)-3 (% cell growth of MO7e cells, 5.9 ± 5.0 % and 1.2 ± 1.1 %, respectively, *p* = 0.17). As shown in Fig. 2c, d, the growth of GMR-negative K562 and Daudi cells was not affected by co-culture with GMR CAR T cells.

### GMR CAR T cells inhibit the colony growth of JMML CD34^+^ cells, but not that of cord blood or bone marrow CD34^+^ Cells

**Fig. 3:**
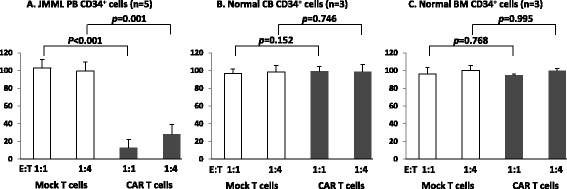
Inhibitory effects of GMR CAR T cells on colony growth of JMML, normal CB, and normal BM CD34^+^ cells. Either GMR CAR T cells or mock T cells were incubated with JMML PB CD34^+^ cells (*n* = 5), normal CB CD34^+^ cells (*n* = 3), or normal BM CD34^+^ cells (*n* = 3) at E:T ratios of 1:1 and 1:4, in the presence of SCF + TPO + IL-3 for 2 days. Cells were then cultured on methylcellulose in media supplemented with GM-CSF, SCF, IL-3, and erythropoietin. After 14 days, the total number of colonies (GM colonies plus erythroid colonies) was calculated for each. Values are expressed as percentages of the total colony numbers obtained by culture in the absence of T cells. The total numbers of colonies grown by 500 JMML PB CD34^+^ cells, CB CD34^+^ cells, and BM CD34^+^ cells in the absence of T cells were 45.4 ± 11.6, 81.3 ± 19.1, and 43.6 ± 0.8, respectively

We evaluated the anti-proliferative effects of GMR CAR T cells on JMML CD34^+^ cells of patients carrying *PTPN11* mutations, *NRAS* mutations, or monosomy 7 (patient nos. 1–5). Either GMR CAR T cells or mock T cells were co-cultured with JMML CD34^+^ cells at E:T ratios of 1:1 and 1:4 in the presence of SCF + TPO + IL-3 for 2 days. Cells were then cultured on methylcellulose in the presence of GM-CSF, SCF, IL-3, and erythropoietin for 14 days. As shown in Fig. 3a, GMR CAR T cells markedly decreased the total number of colonies (granulocyte-macrophage (GM) colonies plus erythroid colonies) by 12.6 ± 9.8 and 28.1 ± 11.3 % at E:T ratios of 1:1 and 1:4, respectively, compared to control cultures. On the other hand, the survival of progenitor cells was observed in co-cultures with mock T cells with total colony numbers of 102.7 ± 10.0 and 99.3 ± 10.1 % at E:T ratios of 1:1 and 1:4, respectively, compared to control cultures. To further investigate whether the GMR CAR T cells expressing GM-CSF as an antigen binding site simulates the proliferation of JMML CD34^+^ cells, we extended the co-culture by 7 days before the methylcellulose colony-forming assay in a case (patient no. 3). The extended co-culture did not lead to an increase in the number of colonies from JMML CD34^+^ cells (the inhibitory effect of GMR CAR T cells, 11.8 % at a 1:1 ratio and 11.8 % at a 1:4 ratio by a 2-day co-culture versus 0.8 % at a 1:1 ratio and 19.3 % at a 1:4 ratio by a 7-day co-culture).

**Fig. 4:**
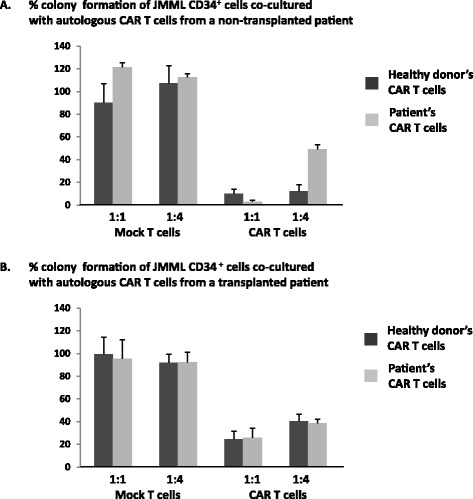
Effects of GMR CAR T cells generated from two patients with JMML on the colony growth of JMML CD34^+^ cells isolated at the time diagnosis. **a** Effect of mock T cells or GMR CAR T cells generated from pre-transplant PBMCs in a patient with an *NRAS* mutation who received no chemotherapy (patient no. 3) on the colony growth of corresponding CD34^+^ cells isolated at disease onset were evaluated. The inhibitory effects of GMR CAR T cells were compared between the patient and a healthy donor. **b** Effects of mock T cells or GMR CAR T cells generated from a patient with both *PTPN11* and *SETBP1* mutations who achieved complete chimerism after cord blood transplantation from an unrelated donor (patient no. 4) on the colony growth of corresponding CD34^+^ cells isolated at disease onset were evaluated. The inhibitory effects of GMR CAR T cells were compared between the patient and a healthy donor. Values are expressed as percentages of the total colony numbers obtained by culture in the absence of T cells. The total numbers of colonies grown by JMML PB CD34^+^ cells in the absence of T cells were 71.3 ± 2.3 in patient no. 3 and 55.3 ± 5.1 in patient no. 4

Next, we evaluated the effect of GMR CAR T cells established from two patients with JMML on the proliferation of corresponding JMML CD34^+^ cells isolated at diagnosis. We generated GMR CAR T cells from pre-transplant PBMCs isolated from a patient with *NRAS*-mutated (38G > A) JMML who received no chemotherapy (patient no. 3). The patient-derived GMR CAR T cells did not harbor the *NRAS* mutation. As presented in Fig. 4a, GMR CAR T cells substantially suppressed the colony growth of corresponding CD34^+^ cells isolated at the time of diagnosis. The anti-proliferative effect at an E:T ratio of 1:1 was almost equivalent between autologous GMR CAR T cells and healthy donor-derived GMR CAR T cells. We then assessed the anti-proliferative ability of GMR CAR T cells generated from a patient with both *PTPN11* and *SETBP1* mutations who achieved complete chimerism following cord blood transplantation from an unrelated donor and had been treated with a small amount of immunosuppressants for chronic graft-versus-host disease (patient no. 4) [4]. As presented in Fig. 4b, the addition of GMR CAR T cells generated from patient no. 4 resulted in an approximately 20 % decrease at an E:T ratio of 1:1, and an approximately 40 % decrease at 1:4, in the proliferation of corresponding JMML CD34^+^ cells isolated at diagnosis compared to culture in the absence of T cells. The inhibitory ability was comparable to that of GMR CAR T cells derived from a healthy donor.

Interestingly, no significant difference in the number of total colonies grown by cord blood (CB) CD34^+^ cells was observed between co-culture with GMR CAR T cells and co-culture with mock T cells. Similarly, the colony growth of bone marrow (BM) CD34^+^ cells was not found to be influenced by the addition of GMR CAR T cells. Moreover, there was no significant effect of GMR CAR T cells on the myelomonocytic and erythroid differentiation of CB CD34^+^ cells or BM CD34^+^ cells. These results are shown in Fig. 3b, c, and Additional file 1: Figure S1.

### Expression of GMR on JMML and CB CD34^+^ cells

**Fig. 5:**
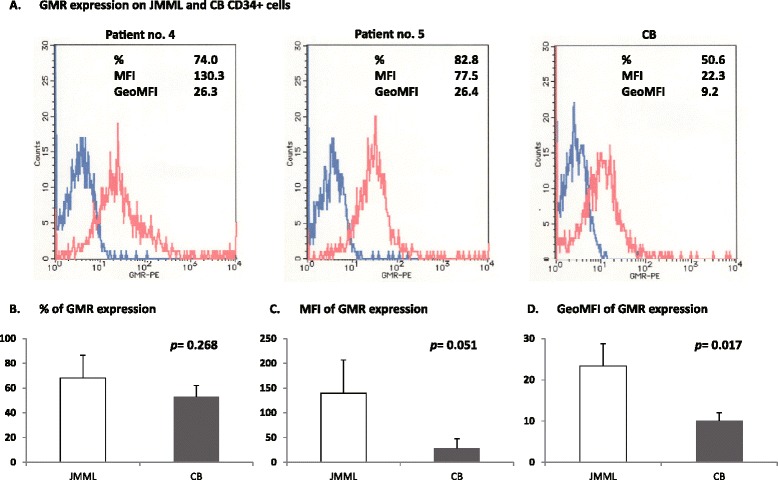
Surface expression of GMR on JMML and CB CD34^+^ cells. **a** Representative results of flow cytometric analysis of GMR expression on JMML CD34^+^ cells and CB CD34^+^ cells. **b** Relative frequency (% positive cells) of GMR expression on JMML CD34^+^ cells (68.17 ± 18.28, *n* = 5) and CB CD34^+^ cells (53.03 ± 9.06, *n* = 3). **b** Mean fluorescence intensity (MFI) of GMR expression on JMML CD34^+^ cells (139.61 ± 67.26, *n* = 5) and CB CD34^+^ cells (28.89 ± 18.62, *n* = 3). **c** Geometric mean fluorescence intensity (GoeMFI) of GMR expression on JMML CD34^+^ cells (23.26 ± 5.36, *n* = 5) and CB CD34^+^ cells (10.05 ± 1.97, *n* = 3)

To investigate the marked difference in response to GMR CAR T cells between JMML CD34^+^ cells and CB CD34^+^ cells, we compared the surface expression of GMR. As shown in Fig. 5, flow cytometric analysis demonstrated greater expression of GMR by JMML CD34^+^ cells (*n* = 5) compared to CB CD34^+^ cells (*n* = 3), according to mean fluorescence intensity (MFI, 139.61 ± 67.26 and 28.89 ± 18.62, respectively, *p* = 0.051) and geometric MFI (23.26 ± 5.36 and 10.05 ± 1.97, respectively, *p* = 0.016). However, no statistical difference in the relative frequency (% positive cells) of GMR expression was observed between JMML CD34^+^ cells and CB CD34^+^ cells (68.17 ± 18.28 and 53.03 ± 9.06, respectively, *p* = 0.268).

### GMR CAR T cells inhibit proliferation of JMML CD34^+^ cells under stimulation with SCF plus TPO on AGM-S3 cells

**Fig. 6:**
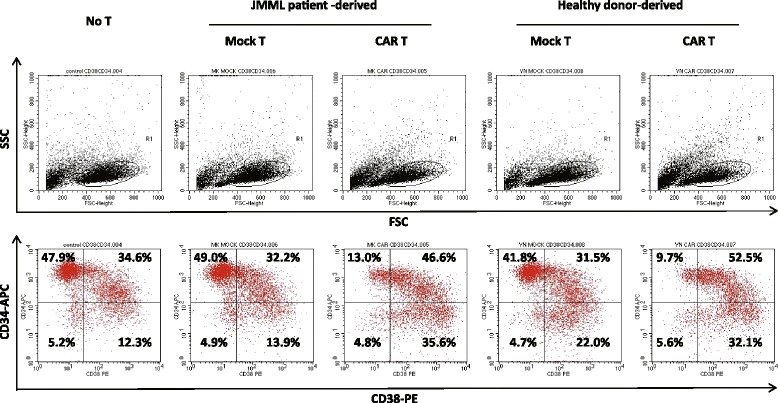
Effects of GMR CAR T cells derived from a patient with JMML and healthy donors on the proliferation of JMML CD34^+^ cell populations. PB CD34^+^ cells (1 × 10^4^) isolated from a patient with *PTPN11* mutation and trisomy 8 (patient no. 6) were cultured on irradiated AGM-S3 cells with or without 1× 10^4^ mock T cells or 1 × 10^4^ GMR CAR T cells in 10 % FBS-containing culture supplemented with 10 ng/ml SCF plus 10 ng/ml TPO. After 7 days, cultured cells were stained with APC-conjugated anti-CD34 and PE-conjugated anti-CD38 mAbs. The addition of both JMML patient-derived GMR CAR T cells and healthy donor-derived GMR CAR T cells reduced the number of JMML CD34^+^CD38^−^ cells compared to culture in the absence of T cells or culture in the presence of mock T cells

Recently, we reported that JMML CD34^+^ cells grown on AGM-S3 cells in the presence of SCF + TPO for 7 days contain leukemic stem cells capable of transplantation into immunodeficient mice and also maintain the comparable expression of GMR [17]. As presented in Table 1, peripheral blood CD34^+^ cells isolated from a patient with monosomy 7 (patient no. 5) [17] were expanded to approximately ninefold the initial number following stimulation with SCF + TPO on irradiated AGM-S3 cells for 7 days. The addition of healthy donor-derived GMR CAR T cells at an E:T ratio of 1:1 resulted in a marked decrease in the number of cultured CD34^+^ cells compared to cultures in the absence of T cells or in the presence of mock T cells. Interestingly, GMR CAR T cells decreased CD34^+^CD38^−^ cell proliferation to a greater extent than that of CD34^+^CD38^+^ cells (Fig. 6 and Table 1). The suppressive potential of GMR CAR T cells derived from a JMML patient who received cord blood transplantation (patient no. 4) was equivalent to that of GMR CAR T cells derived from a healthy donor. On the other hand, mock T cells were found to have no, or a modest, inhibitory effect on the proliferation of CD34^+^ cells, CD34^+^CD38^−^ cells, and CD34^+^CD38^+^ cells. SCF + TPO-dependent proliferation of CD34^+^ cells from a patient with *PTPN11* mutation and trisomy 8 (patient no. 6) cultured on AGM cells was substantially suppressed by the addition of GMR CAR T cells. Meanwhile, the addition of GMR CAR T cells to CB CD34^+^ cell cultures resulted in no significant reductions in the numbers of CD34^+^ cells, CD34^+^CD38^−^ cells, or CD34^+^CD38^+^ cells after 7 days of culture compared with culture in the absence of T cells (data not shown).

**Table 1:** Effects of GMR CAR-T cells on expansion of JMML CD34^+^ cells under stimulation with SCF+TPO on AGM-S3 cells

	None	Healthy T cells		JMML T cells^a^	
		Mock 1:1	CAR 1:1	Mock 1:1	CAR 1:1
Patient no. 5					
CD34^+^ cells (×10^4^)	8.85 ± 0.44	7.75 ± 0.62	2.57 ± 0.33	6.73 ± 0.52	0.88 ± 0.04
CD34^+^CD38^−^ cells (×10^4^)	3.78 ± 0.19	3.20 ± 0.26	0.04 ± 0	2.80 ± 0.22	0 ± 0
CD34^+^CD38^+^ cells (×10^4^)	5.07 ± 0.25	4.56 ± 0.37	2.53 ± 0.33	3.93 ± 0.30	0.88 ± 0.04
Patient no. 6					
CD34^+^ cells (×10^4^)	9.38 ± 0.77	8.15 ± 0.67	1.91 ± 0.17^*^	7.50 ± 0.49	2.50 ± 0.20^*^
CD34^+^CD38^−^ cells (×10^4^)	5.45 ± 0.45	4.65 ± 0.38	0.30 ± 0.03^*^	4.52 ± 0.30	0.55 ± 0.04^*^
CD34^+^CD38^+^ cells (×10^4^)	3.93 ± 0.32	3.50 ± 0.29	1.62 ± 0.15^*^	2.98 ± 0.19	1.96 ± 0.16^*^

*Patient no. 5* monosomy 7, *patient no. 6* trisomy 8 plus *PTPN11* mutation, *GMR* GM-CSF receptor, *CAR* chimeric antigen receptor, *JMML* juvenile myelomonocytic leukemia, *SCF* stem cell factor, *TPO* thrombopoietin

^*^ *p* < 0.0001, significant different from mock T cells

^a^T cells were obtained from patient no. 4 with *PTPN11* and *SETBP1* mutations after unrelated CBT

## Discussion

There is a considerable concern regarding the inhibition of normal myelopoiesis during the development of novel CAR T cell therapies for myeloid malignancies based on the results of preclinical studies of scFv-based CAR T cells targeting CD33 or CD123 antigens [18–21]. Frankel et al. [22] reported that fusion of diphtheria toxin and GM-CSF (DT388-GM-CSF) was toxic to blasts from patients with JMML, whereas normal clonogenic progenitors were insensitive to DT388-GM-CSF. In light of these findings, we generated a GMR-redirected CAR, which encoded GM-CSF as an antigen-binding domain, in the present study. GMR CAR T cells demonstrated substantial cytotoxic effects on MO7e cells expressing GMR in the presence of GM-CSF as well as SCF + TPO + IL-3. On the other hand, the proliferation of GMR-negative K562 and Daudi cells was not found to be affected by the addition of GMR CAR T cells. There was a concern that GMR CAR T cells expressing GM-CSF simulate the proliferation of leukemic cells expressing GMR; however, we found no apparent proliferative effect of GMR CAR T cells on JMML CD34^+^ cells. Therefore, ligand-based GMR CAR T cells may exert a relevant degree of cytolytic activity on leukemic cells expressing GMR.

Recent studies have elucidated genotype-phenotype correlations in JMML [1–6, 23–26]. Patients with *PTPN11* mutations appear to have less favorable outcomes, even after hematopoietic stem cell transplantation; whereas, spontaneous resolution of hematologic abnormalities may occur in a proportion of children with *RAS* or *CBL* mutations. The co-culture of healthy donor-derived GMR CAR T cells with JMML CD34^+^ cells possessing *PTPN11* mutations, *NRAS* mutations, or monosomy 7 for 2 days resulted in a significant reduction in the total number of colonies compared to co-culture with mock T cells. Similarly, autologous GMR CAR T cells were found to inhibit the proliferation of corresponding CD34^+^ cells isolated at disease onset to a degree comparable with GMR CAR T cells generated from a healthy donor. Importantly, GMR CAR T cells were found to have no effect on the colony growth and the myelomonocytic differentiation of CB CD34^+^ cells or BM CD34^+^ cells. Therefore, GMR CAR T cells appear to have substantial cytotoxic effects on JMML progenitors, regardless of genetic abnormality, but not on normal hematopoietic progenitors. An equivalent degree of anti-leukemic activity was observed in GMR CAR T cells established from a JMML patient who had been treated with immunosuppressants following cord blood transplantation (patient no. 4) compared to GMR CAR T cells generated from a healthy donor. Thus, adoptive immunotherapy demonstrated in this study may have utility in the treatment of post-transplant relapse in cases of JMML.

There was a significant discrepancy in the responses of JMML CD34^+^ cells and normal CD34^+^ cells to GMR CAR T cells. According to flow cytometric analyses, GMR expression by JMML CD34^+^ cells, determined by geometric MFI, was almost twofold higher than by normal CD34^+^ cells; whereas, no significant difference in GMR expression determined by the relative frequency was observed between the two cell types. It is demonstrated that GMR forms a unique and distinctive crystal structure during activation: a low-affinity complex consisting of GM-CSF bound to a GMR α subunit (GMRα), i.e., one GM-CSF and one GMRα; a high-affinity hexamer complex formed by interaction with multiple GMR βc subunits (βc), i.e., two GM-CSF, two GMRα, and two βc; and a dodecamer complex formed by lateral aggregation of hexamer complexes, i.e., four GM-CSF, four GMRα, and four βc [27, 28]. Dodecamer formation appears to be essential for receptor activation and subsequent signal transduction. Given this model of GMR activation [27, 28], susceptibility to ligand-based GMR CAR T cell-mediated cytotoxicity may be mediated, in part, by differing GMR complex compositions in JMML CD34^+^ cells and CB CD34^+^ cells, although we have no direct evidence that JMML CD34^+^ cells possess a hexamer or higher-order complex of GMR.

We previously reported that stimulation of JMML CD34^+^ cells with SCF + TPO during culture on AGM-S3 cells led to substantial expansion of JMML CD34^+^ cells that contained leukemic stem cells capable of transplantation into immunodeficient mice after 7 days culture [17]. In the present study, we found that the addition of GMR CAR T cells to cultures of CD34^+^ cells from a patient with monosomy 7 (patient no. 5) resulted in marked inhibition of the proliferation of CD34^+^ cells, particularly CD34^+^CD38^−^ cells, stimulated with SCF + TPO on AGM-S3 cells. GMR CAR T cells were also found to have substantial anti-proliferative activity on CD34^+^ cells with mutated *PTPN11* and trisomy 8. These results indicate that GMR CAR T cells developed in this study display cytolytic effects on both leukemic stem cells and progenitor cells isolated from patients with JMML.

## Conclusions

Ligand-based GMR CAR T cells may have leukemic activity against JMML stem and progenitor cells, regardless of genetic abnormality, but not against normal hematopoietic stem cells.

## Methods

This study was conducted in accordance with the Helsinki Declaration and was approved by the institutional review board of Shinshu University School of Medicine.

### Plasmids

The *piggyBac* transposase plasmid (pCMV-*piggyBac*) and the *piggyBac* transposon plasmid for CD19 CAR (pIRII-CAR.CD19) have been described previously [15, 16]. We constructed a novel *piggyBac* transposon plasmid to express a CAR targeting GMR (pIRII-CAR.GMR) by replacing the coding region of the anti-CD19 scFv in pIRII-CAR.CD19 with the open reading frame of the GM-CSF cDNA (OirGene Technologies, Inc., MD). The pIRII-CAR.GMR is transcriptionally regulated by a cytomegalovirus immediate early gene enhancer/promoter sequence and encodes GM-CSF, linked to the ch2ch3 domain of human IgG1, the endodomains of CD28, and a T-cell receptor ζ chain (Fig. 1a). Plasmid constructs were confirmed by restriction digestion and DNA sequencing.

### Cell preparation

To generate GMR CAR-modified T cells, PBMCs were obtained from three healthy adult volunteers and two patients with JMML. Informed consent was obtained from volunteers and patient guardians, respectively.

JMML CD34^+^ cells were enriched from PBMCs frozen at the time of disease onset from six patients by positive immunomagnetic selection using a CD34 MicroBead kit (Miltenyi Biotec, Inc., Auburn, CA). The following gene mutations were detected in JMML CD34^+^ cells: patient no. 1 [26], *NRAS* 34G > A; patient no. 2, *PTPN11* 227A > G; patient no. 3, *NRAS* 38G > A; patient no. 4 [4], *PTPN11* 182A > T and *SETBP1* D868N; patient no. 5 [17], monosomy 7; and patient no. 6, *PTPN11* 227A > G and trisomy 8. Flow cytometric analysis revealed that 99 % of isolated cells were CD34^+^. CB CD34^+^ cells and BM CD34^+^ cells were purchased from Riken BioResource Center (Tsukuba, Japan) and STEMCELL Technologies, Inc. (Vancouver, Canada), respectively.

An acute megakaryoblastic leukemia cell line, MO7e, was obtained from Genetics Institute (Boston, MA) and maintained in RPMI 1640 medium (Life Technologies, Co. Ltd., Carlsbad, CA) supplemented with 10 % fetal bovine serum (FBS, HyClone Laboratories, Inc., Logan, UT) in the presence of recombinant human GM-CSF (10 ng/mL, Miltenyi Biotec). A chronic myeloid leukemia cell line, K562, was purchased from Riken BioResource Center and maintained in 10 % FBS containing RPMI 1640 medium without the addition of cytokines. A Burkitt’s lymphoma cell line, Daudi, was purchased from ATCC (Manassas, VA) and maintained in 10 % FBS containing RPMI 1640 medium.

### Flow cytometric analysis

Using the BD FACSCalibur with BD CellQuest Pro software (Becton, Dickinson and Company (BD), Franklin Lakes, NJ), we evaluated the antigen profile of expanded CAR T cells, MO7e, K562, and Daudi cell lines, and cultured cells generated from JMML and CB CD34^+^ cells. Allophycocyanin (APC)-conjugated anti-CD3 mAb, phycoerythrin (PE)-conjugated anti-CD4 mAb, and APC-conjugated anti-CD8 mAb (Myltenyi Biotec) were used to stain expanded CAR T cells. CAR expression by T cells was determined by staining with APC-conjugated anti-CD3 mAb and fluorescein isothiocyanate (FITC)-conjugated goat anti-human IgG (H + L) (Jackson ImmunoResearch Laboratories, Inc., West Grave, PA). GMR expression on JMML and CB CD34^+^ cells was determined using PE-conjugated anti-CD116 mAb (BD) and APC-conjugated anti-CD34 mAb (Myltenyi Biotec). APC-conjugated anti-CD3 mAb and PE-conjugated anti-CD33 mAb (BD) were used to evaluate co-cultures of GMR CAR T cells with MO7e or K562 cells. APC-conjugated anti-CD3 mAb and PE-conjugated anti-CD19 mAb (Myltenyi Biotec) were used to evaluate co-cultures of GMR CAR T cells with Daudi cells. APC-conjugated anti-CD34 mAb and PE-conjugated anti-CD38 mAb (BD) were used to evaluate co-cultures of GMR CAR T cells with JMML or CB CD34^+^ cells. APC-, FITC-, and PE-conjugated mouse isotype-matched IgG (Myltenyi Biotec or BD) were used as controls in all experiments.

### Gene transfer into T cells and expansion of transgenic T cells

We previously reported non-viral gene transfer of the CAR gene into T cells and the efficient expansion of CAR-modified T cells using *piggyBac* transposons in a serum-free culture system [15]. Briefly, unstimulated PBMCs obtained from 10 ml peripheral blood were electroporated with a pIRII-CAR.GMR plasmid (5 μg) and a pCMV-*piggyBac* plasmid (5 μg) using a 4D-Nucleofector Device (Program EO-115) and P3 Primary Cell 4D-Nucleofector X Kits (Lonza, Basel, Switzerland). Transgenic cells were maintained in serum- and xeno-free T cell culture medium (TexMACS Medium, Miltenyi Biotec) supplemented with recombinant human IL-15 (5 ng/ml, Miltenyi Biotec) at 37 °C in a humidified 5 % CO_2_ incubator. On the following day, cells were transferred and cultured in 24-well culture plates coated with anti-CD3 mAb and anti-CD28 mAb (Miltenyi Biotec). Six days after stimulation, cells were labeled with biotin-conjugated goat anti-human IgG (H + L) (Jackson ImmunoResearch) with affinity for the IgG1 ch2ch3 and selected based on CAR expression using Anti-Biotin MicroBeads (Miltenyi Biotec) and a MACS Column (Miltenyi Biotec). Negatively selected cells (almost entirely consisting of CAR-negative activated T cells) were irradiated and plated as feeder cells. Positively selected cells were re-stimulated in anti-CD3/CD28 mAbs-coated wells with autologous feeder cells in TexMACS medium containing 5 ng/ml of IL-15 for 4 days before transfer to a G-Rex 10 device (Wilson Wolf Manufacturing, Inc., New Brighton, MN) with 30 ml of IL-15-containing TexMACS medium for a further 10 days. IL-15-containing TexMACS medium was half changed every 4 to 5 days during the culture period. The number of viable cells was determined by the trypan blue exclusion test using a hemocytometer. After 21 days of culture, cells were cryopreserved at −80 °C for use in subsequent experiments. Non-electroporated PBMCs concurrently stimulated in anti-CD3/CD28 mAbs-coated plates and cultured in IL-15-containing TexMACS medium for 21 days were used as controls (mock T cells).

### Co-culture of GMR CAR T cells with MO7e, K562, or Daudi cells

The anti-proliferative effects of GMR CAR T cells on leukemia/lymphoma cells were evaluated using co-culture experiments with three cell lines: MO7e cells express GMR on the cell surface, while K562 and Daudi cells do not. Briefly, either GMR CAR T cells or mock T cells were added to 2 × 10^5^ MO7e, K562, or Daudi cells at an E:T ratio of 2:1 in 48-well flat-bottom culture plate wells containing RPMI 1640 medium plus 10 % FBS. In MO7e cell cultures, a combination of recombinant human SCF (Miltenyi Biotec), TPO (Miltenyi Biotec), and IL-3 (Miltenyi Biotec) or GM-CSF was added to the culture medium. MO7e, K562, and Daudi cells were cultured alone in identical conditions and used as controls. After 4 days, the number of viable cells was determined by the trypan blue exclusion test and the percentages of CAR T cells and leukemia/lymphoma cells were determined by flow cytometry using APC-conjugated anti-CD3 mAb and either of PE-conjugated anti-CD33 mAb or PE-conjugated anti-CD19 mAb, respectively.

### Evaluation of the anti-proliferative effects of GMR CAR T cells on JMML, CB, and BM CD34^+^ cells

To examine the anti-proliferative effects of GMR CAR T cells on JMML and normal CD34^+^ cells, we performed co-cultures and subsequent clonal cell cultures according to modification of a previously described method [15, 17, 29]. Briefly, either GMR CAR T cells or mock T cells were added to 500 JMML, CB, or BM CD34^+^ cells at an E:T ratio of 1:1 or 1:4 in 96-well round-bottom plates (Nalge Nunc International, NY) containing RPMI 1640 medium plus 10 % FBS in the presence of 10 ng/ml SCF, 10 ng/ml TPO, and 10 ng/ml IL-3. As controls, CD34^+^ cells were cultured alone in identical conditions. After 2 days, cultured cells were plated in 35-mm Lux suspension culture dishes (Nalge Nunc International) containing methylcellulose media supplemented with GM-CSF, SCF, IL-3, and erythropoietin (Methocult GF H4434, STEMCELL Technlogies). Dishes were incubated at 37 °C in a humidified atmosphere containing 5 % CO_2_. On day 14, the total numbers of GM and erythroid colonies were scored in situ on an inverted microscope as both colony types were derived from single malignant clones [23, 30].

### Suspension culture of JMML CD34^+^ cells

As described previously [16], 1 × 10^4^ JMML or CB CD34^+^ cells were cultured with 1 × 10^4^ GMR CAR T cells or mock T cells in 10 % FBS-containing MEM alpha medium (Life Technologies) supplemented with 10 ng/ml SCF plus 10 ng/ml TPO on irradiated, confluent AGM-S3 cells (kindly provided by Kyowa Hakko Kirin, Co. Ltd., Tokyo, Japan) in 35-mm gelatin-coated dishes. As controls, CD34^+^ cells were cultured alone in identical conditions. After 7 days, the number of viable cells was determined by the trypan blue exclusion test. To evaluate the antigenic profile of cultured cells, 1 to 2 × 10^5^ cells were then collected in plastic tubes and incubated with a combination of appropriately diluted APC-conjugated anti-CD34 mAb and PE-conjugated anti-CD38 mAb.

### Statistical analyses

All values are expressed as mean ± SD. To determine the significance of difference between two independent groups, we used the pared or unpaired *t* test. The chi-squared test was used to compare the anti-leukemic activity of GMR CAR T cells between patients and healthy donors. Statistical significance was defined as *p* < 0.05.

## Additional file

Additional file 1: Figure S1. Effects of GMR CAR T cells on GM or erythroid colony growth of CB and BM CD34^+^ cells. Either GMR CAR T cells or mock T cells were incubated with normal CB CD34^+^ cells (*n* = 3) or normal BM CD34^+^ cells (*n* = 3) at E:T ratios of 1:1 and 1:4, in the presence of SCF + TPO + IL-3 for 2 days. Cells were then cultured on methylcellulose in media supplemented with GM-CSF, SCF, IL-3, and erythropoietin. After 14 days, the numbers of GM colonies and erythroid colonies were calculated for each. Values are expressed as percentages of the total colony numbers obtained by culture in the absence of T cells. The numbers of GM colonies grown by 500 CB CD34^+^ cells and BM CD34^+^ cells in the absence of T cells were 38.6 ± 4.6 and 24.5 ± 2.9, respectively. The numbers of erythroid colonies grown by 500 CB CD34^+^ cells and BM CD34^+^ cells in the absence of T cells were 42.7 ± 15.5 and 19.1 ± 2.6, respectively. (EPS 439 kb)

## Abbreviations

APC: allophycocyanin
BM: bone marrow
CAR: chimeric antigen receptors
CB: cord blood
E:T: effector to target
FBS: fetal bovine serum
FITC: fluorescein isothiocyanate
GM-CSF: granulocyte-macrophage colony-stimulating factor
GMR: granulocyte-macrophage colony-stimulating factor receptor
IL: interleukin
JMML: juvenile myelomonocytic leukemia
mAb: monoclonal antibody
PBMCs: peripheral blood mononuclear cells
PE: phycoerythrin
SCF: stem cell factor
scFv: single-chain variable fragment
TPO: thrombopoietin

## References

[1] Koike K, Matsuda K (2008). Recent advances in the pathogenesis and management of juvenile myelomonocytic leukemia. Br J Haematol.

[2] Dvorak CC, Loh ML (2014). Juvenile myelomonocytic leukemia: molecular pathogenesis informs current approaches to therapy and hematopoietic cell transplantation. Front Pediatr.

[3] Sakaguchi H, Okuno Y, Muramatsu H, Yoshida K, Shiraishi Y, Takahashi M (2013). Exome sequencing identifies secondary mutations of SETBP1 and JAK3 in juvenile myelomonocytic leukemia. Nat Genet.

[4] Matsuda K, Nakazawa Y, Iwashita C, Kurata T, Hirabayashi K, Saito S (2014). Myeloid progenitors with PTPN11 and nonRAS pathway gene mutations are refractory to treatment with 6-mercaptopurine in juvenile myelomonocytic leukemia. Leukemia.

[5] Stieglitz E, Troup CB, Gelston LC, Haliburton J, Chow ED, Yu KB (2015). Subclonal mutations in SETBP1 confer a poor prognosis in juvenile myelomonocytic leukemia. Blood.

[6] Caye A, Strullu M, Guidez F, Cassinat B, Gazal S, Fenneteau O (2015). Juvenile myelomonocytic leukemia displays mutations in components of the RAS pathway and the PRC2 network. Nat Genet.

[7] Locatelli F, Niemeyer CM (2015). How I treat juvenile myelomonocytic leukemia. Blood.

[8] Worth A, Rao K, Webb D, Chessells J, Passmore J, Veys P (2003). Successful treatment of juvenile myelomonocytic leukemia relapsing after stem cell transplantation using donor lymphocyte infusion. Blood.

[9] Yoshimi A1, Bader P, Matthes-Martin S, Starý J, Sedlacek P, Duffner U (2005). Donor leukocyte infusion after hematopoietic stem cell transplantation in patients with juvenile myelomonocytic leukemia. Leukemia.

[10] Nabarro S, Thrasher AJ, Kempski H, Amrolia P, Anderson J (2003). Generation of immunostimulatory dendritic cells from the malignant clone in patients with juvenile myelomonocytic leukemia. Leukemia.

[11] Hirano N, Butler MO, Xia Z, Berezovskaya A, Murray AP, Ansén S (2006). Identification of an immunogenic CD8+ T-cell epitope derived from gamma-globin, a putative tumor-associated antigen for juvenile myelomonocytic leukemia. Blood.

[12] Davila ML, Bouhassira DC, Park JH, Curran KJ, Smith EL, Pegram HJ (2014). Chimeric antigen receptors for the adoptive T cell therapy of hematologic malignancies. Int J Hematol.

[13] Maude SL, Frey N, Shaw PA, Aplenc R, Barrett DM, Bunin NJ (2014). Chimeric antigen receptor T cells for sustained remissions in leukemia. N Engl J Med.

[14] Lee DW, Kochenderfer JN, Stetler-Stevenson M, Cui YK, Delbrook C, Feldman SA (2015). T cells expressing CD19 chimeric antigen receptors for acute lymphoblastic leukemia in children and young adults: a phase 1 dose-escalation trial. Lancet.

[15] Saito S, Nakazawa Y, Sueki A, Matsuda K, Tanaka M, Yanagisawa R (2014). Anti-leukemic potency of piggyBac-mediated CD19-specific T cells against refractory Philadelphia chromosome-positive acute lymphoblastic leukemia. Cytotherapy.

[16] Huye LE, Nakazawa Y, Patel MP, Yvon E, Sun J, Savoldo B (2011). Combining mTor inhibitors with rapamycin-resistant T cells: a two-pronged approach to tumor elimination. Mol Ther.

[17] Sakashita K, Kato I, Daifu T, Saida S, Hiramatsu H, Nishinaka Y (2015). In vitro expansion of CD34^+^CD38^−^ cells under stimulation with hematopoietic growth factors on AGM-S3 cells in juvenile myelomonocytic leukemia. Leukemia.

[18] Marin V, Pizzitola I, Agostoni V, Attianese GM, Finney H, Lawson A (2010). Cytokine-induced killer cells for cell therapy of acute myeloid leukemia: improvement of their immune activity by expression of CD33-specific chimeric receptors. Haematologica.

[19] Mardiros A, Dos Santos C, McDonald T, Brown CE, Wang X, Budde LE (2013). T cells expressing CD123-specific chimeric antigen receptors exhibit specific cytolytic effector functions and antitumor effects against human acute myeloid leukemia. Blood.

[20] Pizzitola I, Anjos-Afonso F, Rouault-Pierre K, Lassailly F, Tettamanti S, Spinelli O (2014). Chimeric antigen receptors against CD33/CD123 antigens efficiently target primary acute myeloid leukemia cells in vivo. Leukemia.

[21] Gill S, Tasian SK, Ruella M, Shestova O, Li Y, Porter DL (2014). Preclinical targeting of human acute myeloid leukemia and myeloablation using chimeric antigen receptor-modified T cells. Blood.

[22] Frankel AE, Lilly M, Kreitman R, Hogge D, Beran M, Freedman MH (1998). Diphtheria toxin fused to granulocyte-macrophage colony-stimulating factor is toxic to blasts from patients with juvenile myelomonocytic leukemia and chronic myelomonocytic leukemia. Blood.

[23] Matsuda K, Shimada A, Yoshida N, Ogawa A, Watanabe A, Yajima S (2007). Spontaneous improvement of hematologic abnormalities in patients having juvenile myelomonocytic leukemia with specific RAS mutations. Blood.

[24] Yoshida N, Yagasaki H, Xu Y, Matsuda K, Yoshimi A, Takahashi Y (2009). Correlation of clinical features with the mutational status of GM-CSF signaling pathway-related genes in juvenile myelomonocytic leukemia. Pediatr Res.

[25] Niemeyer CM, Kang MW, Shin DH, Furlan I, Erlacher M, Bunin NJ (2010). Germline CBL mutations cause developmental abnormalities and predispose to juvenile myelomonocytic leukemia. Nat Genet.

[26] Matsuda K, Yoshida N, Miura S, Nakazawa Y, Sakashita K, Hyakuna N (2012). Long-term hematological improvement after non-intensive or no chemotherapy in juvenile myelomonocytic leukemia and poor correlation with adult myelodysplasia spliceosome-related mutations. Br J Haematol.

[27] Hansen G, Hercus TR, McClure BJ, Stomski FC, Dottore M, Powell J (2008). The structure of the GM-CSF receptor complex reveals a distinct mode of cytokine receptor activation. Cell.

[28] Hercus TR, Thomas D, Guthridge MA, Ekert PG, King-Scott J, Parker MW (2009). The granulocyte-macrophage colony-stimulating factor receptor: linking its structure to cell signaling and its role in disease. Blood.

[29] Sawai N, Koike K, Ito S, Mwamtemi HH, Kurokawa Y, Kinoshita T (1999). Neutrophilic cell production by combination of stem cell factor and thrombopoietin from CD34^+^ cord blood cells in long-term serum-deprived liquid culture. Blood.

[30] Matsuda K, Taira C, Sakashita K, Saito S, Tanaka-Yanagisawa M, Yanagisawa R (2010). Long-term survival after nonintensive chemotherapy in some juvenile myelomonocytic leukemia patients with CBL mutations, and the possible presence of healthy persons with the mutations. Blood.

